# Artificial Intelligence-Driven Advances in Coronary Calcium Scoring: Expanding Preventive Cardiology

**DOI:** 10.7759/cureus.74681

**Published:** 2024-11-28

**Authors:** Deepak Dev Vivekanandan, Nikita Singh, Marshall Robaczewski, Abigayle Wyer, Lucas N Canaan, Daniel Whitson, Nathaniel Grabill, Mena Louis

**Affiliations:** 1 General Surgery, Northeast Georgia Medical Center Gainesville, Gainesville, USA; 2 Internal Medicine, Albert Einstein College of Medicine, Jacobi Medical Center, Bronx, USA

**Keywords:** ai in medical imaging, artificial intelligence (ai), cad: coronary artery disease, coronary artery calcium score, nongated chest ct, preventive cardiology

## Abstract

Coronary artery disease (CAD) remains a leading global cause of morbidity and mortality, underscoring the need for effective cardiovascular risk stratification and preventive strategies. Coronary artery calcium (CAC) scoring, traditionally performed using electrocardiogram (ECG)-gated cardiac computed tomography (CT) scans, has been widely validated as a robust tool for assessing cardiovascular risk. However, its application has been largely limited to high-risk populations due to the costs, technical requirements, and limited accessibility of cardiac CT scans. Recent advancements in artificial intelligence (AI) have introduced transformative opportunities to extend CAC detection to noncardiac CT scans, such as those performed for lung cancer screening, enabling broader and more accessible cardiovascular screening.

This review provides a comprehensive analysis of AI-driven CAC detection, examining various types of AI models for CAC detection, like convolutional neural networks (CNNs) and U-Net architectures, and exploring the clinical, operational, and ethical implications of incorporating these technologies into routine practice. Technical challenges, including imaging variability, data privacy, and model bias, are discussed alongside essential areas for further research, such as standardization and validation across diverse populations. By leveraging widely available imaging data, AI-enabled CAC detection has the potential to advance preventive cardiology, supporting earlier risk identification, optimizing healthcare resources, and improving patient outcomes.

## Introduction and background

Coronary artery disease (CAD) remains one of the leading global causes of morbidity and mortality, responsible for a substantial burden on healthcare systems worldwide [1-3]. The increasing incidence of CAD and an aging population underscore the urgent need for effective risk assessment and early preventive measures [4]. Traditional CAD screening methods focus on established risk factors, including lipid profiles, blood pressure, and lifestyle factors [5], which, while valuable, do not provide a direct measure of subclinical atherosclerosis. Coronary artery calcium (CAC) scoring has emerged as a critical marker for assessing cardiovascular risk, providing a quantitative assessment of calcified plaque burden within the coronary arteries [6].

Historically, CAC scoring was developed as a noninvasive tool to assess atherosclerotic disease, initially identified on computed tomography (CT) scans in the 1980s [7]. Through advancements in electrocardiogram (ECG)-gated, contrast-enhanced cardiac CT imaging, CAC scoring has evolved into a validated tool for predicting CAD risk, used in clinical practice for patients undergoing specific cardiac evaluations [8,9]. CAC scores have been shown to correlate strongly with cardiovascular events, and they are widely used to inform preventive strategies, such as statin and aspirin initiation [10].

Despite its utility, traditional CAC scoring via dedicated cardiac CT scans has limitations. Due to the need for ECG gating, contrast enhancement, and the associated costs [11], it is often reserved for high-risk patients or those with symptomatic concerns. This restricts CAC scoring primarily to cardiac-focused settings, leaving a vast portion of the population, especially those undergoing noncardiac imaging, without access to this valuable risk stratification tool.

In the recent years, advances in artificial intelligence (AI) have introduced an innovative approach to CAC detection that could expand the reach of cardiovascular risk assessment. AI-driven algorithms, particularly convolutional neural networks (CNNs) and U-Net architectures, have demonstrated promising accuracy in identifying CAC on noncardiac CT scans [12]. This development represents a significant shift in preventive cardiology, as noncardiac CT scans, such as those used in lung cancer screenings, are performed routinely and provide an untapped resource for incidental CAC detection [13]. By leveraging AI to analyze these noncardiac scans, healthcare providers can opportunistically identify at-risk individuals without additional imaging appointments or radiation exposure, thereby enhancing access to cardiovascular screening [14].

This review will explore the technical, clinical, and operational aspects of AI-driven CAC detection on noncardiac CT scans, addressing the potential for AI to enhance preventive cardiology. We will discuss the technical advancements and challenges inherent in this approach, ethical and legal considerations, and areas requiring further research. Ultimately, AI-driven CAC detection holds the promise of transforming cardiovascular risk stratification, enabling broader and more cost-effective screening and fostering earlier interventions that could improve patient outcomes and alleviate healthcare disparities.

## Review

AI has transformed diagnostic imaging, enabling rapid and accurate analysis of medical images with minimal human oversight. In recent years, advancements in AI, particularly in deep learning, have facilitated the development of models that can detect and quantify CAC on noncardiac CT scans. AI-based models like CNNs and U-Net architectures have proven highly effective at identifying calcifications in noncontrast, non-ECG-gated CT images, thereby extending CAC scoring to a broader range of clinical contexts [12,14].

Advances in AI for CAC detection

Types of AI Models Used for CAC Detection

AI models for CAC detection primarily utilize CNNs and U-Net-based frameworks due to their strong performance in image recognition and segmentation tasks [12]. CNNs, with their layered structure, can capture intricate spatial patterns, making them particularly useful for identifying small, dense areas of calcification within CT images [15,16]. U-Net models, initially designed for biomedical image segmentation, are especially suited for distinguishing calcifications from surrounding anatomical structures [17,18]. U-Net models employ an encoder-decoder architecture, which enables both localization and segmentation of CAC within the cardiac chambers [19].

In addition to CNNs and U-Net models, more advanced architectures like convolutional long short-term memory (conv-LSTM) networks and hybrid models combining CNNs with recurrent neural networks (RNNs) have shown potential for enhancing detection accuracy [20]. Conv-LSTM models incorporate temporal and spatial information, which is advantageous in cases where multiple CT slices must be analyzed sequentially to locate CAC accurately [21]. These models are beneficial in environments where multiple imaging planes or larger anatomical fields are considered.

Additional AI Models for CAC Detection

Beyond CNN, U-Net, and conv-LSTM, recent advancements have introduced other AI architectures that bring unique CAC detection and quantification capabilities, expanding on the foundational deep learning models. These additional models bring unique strengths to the field of CAC detection.

Fully convolutional networks (FCNs): FCNs are optimized for pixel-wise segmentation, which is essential for accurate CAC detection in CT scans. Unlike models with fully connected layers, FCNs consist solely of convolutional layers, making them more efficient for tasks requiring precise boundary delineation. This approach allows FCNs to achieve high sensitivity and specificity in identifying calcified regions, providing detailed segmentation that is crucial for accurate CAC scoring [22].

Deep learning ensembles: Ensemble methods enhance the performance of CAC detection systems by combining multiple models [23]. Techniques like bagging, boosting, and stacking can balance out individual model weaknesses and increase robustness in complex or ambiguous cases [24].

Explainable AI models: The integration of explainable AI models is especially valuable in CAC detection. These models provide interpretability, allowing clinicians to understand the AI’s decision-making process. By providing visual insights, such as heatmaps, these models can enhance clinicians’ trust, ensure alignment with clinical assessments, and promote the adoption of AI tools in routine healthcare practice [25].

Transfer learning approaches: Transfer learning enables AI models to leverage knowledge from large datasets and apply it to specialized tasks like CAC detection. By pretraining on extensive datasets and fine-tuning for CAC detection, transfer learning reduces training time and improves performance, especially when labeled data for CAC is limited [26].

Hybrid models: Hybrid models combine traditional image processing methods with AI-based techniques to enhance CAC detection. They leverage the strengths of both approaches: the precision of classic image processing and the adaptability of deep learning. Hybrid models are particularly effective in cases where images have high noise or ambiguous calcifications, offering a balanced and accurate approach to CAC quantification [27].

Comparison of AI Model Features in CAC Detection

**Figure 1:**
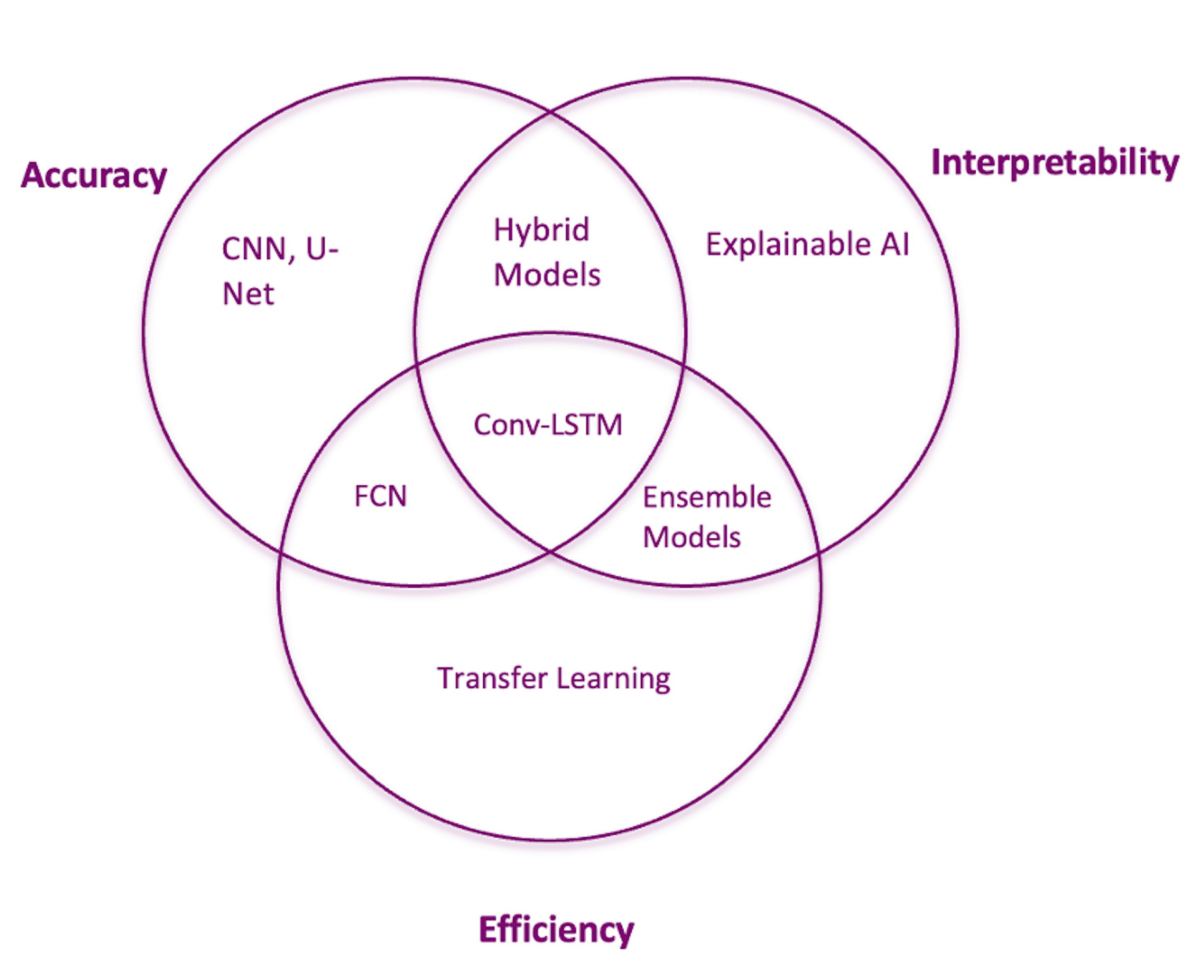
Comparison of AI model features in CAC detection, highlighting strengths in accuracy, interpretability, and efficiency. Models like CNN and U-Net excel in accuracy, FCNs prioritize efficiency, while conv-LSTM balances across all three attributes AI: artificial intelligence; CAC: coronary artery calcium; CNN: convolutional neural network; FCNs: fully convolutional networks; conv-LSTM: convolutional long short-term memory

The following diagram illustrates the primary strengths of various AI models in CAC detection, highlighting where models like CNN, U-Net, and conv-LSTM align in terms of accuracy, interpretability, and efficiency (Figure 1).

Clinical implications and pathway integration

AI-driven CAC detection on noncardiac CT scans has significant potential to reshape cardiovascular risk assessment by allowing clinicians to identify at-risk patients through routine imaging, facilitating early intervention. Studies have demonstrated that incidental CAC detection enhances preventive care, especially in asymptomatic populations and high-risk groups, while minimizing workflow disruptions when carefully integrated into clinical practice [28,29].

Clinical Benefits and Early Risk Stratification

The predictive value of CAC for cardiovascular events is well-established. In the Multi-Ethnic Study of Atherosclerosis (MESA), researchers found that CAC scores strongly correlate with cardiovascular events, underscoring the role of CAC in stratifying risk across diverse ethnic groups [30]. Similarly, a study by Silverman et al. demonstrated that individuals without traditional cardiovascular risk factors with detectable CAC had significantly higher cardiovascular event rates, which supports the use of CAC detection for guiding preventive interventions like lifestyle modifications or statin therapy [31]. AI-driven CAC detection on noncardiac CT scans allows clinicians to opportunistically screen broader populations, including those who may not otherwise undergo dedicated cardiac imaging, thereby extending the reach of early cardiovascular risk assessment.

Applications in High-Risk Populations

Certain high-risk groups, such as smokers undergoing lung cancer screening, can particularly benefit from AI-based CAC detection. In a study by Park et al., CAC detection on lung CT scans showed promise for providing dual benefits: assessing both lung cancer risk and cardiovascular risk in a single imaging session. The study found that patients with high CAC scores identified incidentally were at an elevated risk of cardiovascular events, illustrating the clinical value of integrating CAC assessment into routine screenings for high-risk individuals [32].

Another key population includes individuals with autoimmune diseases, where systemic inflammation elevates cardiovascular risk. A study by Weber et al. demonstrated the utility of AI in identifying CAC in patients with autoimmune disorders, such as lupus and rheumatoid arthritis, showing that over 50% had detectable CAC, with the highest prevalence in those with psoriasis [28]. This study highlights AI’s potential to provide preventive care benefits to specialized populations with elevated cardiovascular risk profiles.

Workflow Integration and Notification Systems

The integration of AI-driven CAC detection into clinical workflows must be seamless to ensure it does not overburden healthcare providers. The Incidental Coronary Calcification Quality Improvement Project (NOTIFY-1) trial offers valuable insights into this aspect. In this trial, automated notifications of incidental CAC findings were sent to primary care providers, prompting timely follow-up without placing additional demands on radiologists or primary care teams [29]. Automated notification systems can help ensure that CAC findings are acted upon promptly, reducing the risk of overlooked incidental findings and enabling clinicians to deliver targeted preventive care to patients with significant CAC.

AI-driven CAC detection can also operate as a background task in radiology workflows. Models analyze CT scans automatically and alert providers if significant calcifications are detected [33]. This approach streamlines workflow efficiency and reduces the need for dedicated cardiac imaging, especially in resource-limited settings where access to specialized cardiac imaging may be limited. Studies exploring the integration of AI into clinical workflows have found that this collaborative approach between AI and radiologists enhances diagnostic precision while maintaining manageable workloads for radiologists [34,35].

Reducing False Positives and Enhancing Model Specificity

While AI-driven CAC detection has shown high sensitivity, minimizing false positives remains essential for practical implementation. False positives, where calcifications outside the coronary arteries, noise, or artifacts are misidentified as CAC [36], can lead to unnecessary follow-up imaging and healthcare costs. Dual-AI frameworks, where one model detects calcifications and a secondary model verifies anatomical location, have shown promise in improving specificity [37].

Ethical, legal, and social implications

While AI-driven CAC detection offers promising benefits for preventive cardiology, it also raises ethical, legal, and social challenges. These challenges center around data privacy, potential biases in AI models, and regulatory considerations necessary for responsible and equitable AI integration into healthcare systems. Addressing these issues is crucial to ensure the safe, fair, and effective use of AI in cardiovascular risk assessment [38,39].

Data Privacy and Security Concerns

AI models rely heavily on large datasets for training, testing, and validation, often involving patient-specific medical images and health records [40]. Given the sensitivity of this data, ensuring proper data privacy and security is paramount. Health data is governed by strict privacy regulations, such as the Health Insurance Portability and Accountability Act (HIPAA) in the United States [41] and the General Data Protection Regulation (GDPR) in the European Union [42]. Compliance with these frameworks requires rigorous data de-identification, secure storage, and clear patient consent protocols.

A study by Rieke et al. on privacy-preserving AI in medical imaging highlights techniques like federated learning, where AI models are trained on decentralized data without transferring it to a central server, thereby preserving privacy. In federated learning, each institution retains patient data locally and only shares the model updates, minimizing the risk of data breaches. Implementing federated learning in CAC detection could enable multicenter collaboration without compromising patient privacy, though logistical challenges remain [43,44].

Bias and Fairness in AI Models

Bias in AI models presents a significant ethical concern, especially in healthcare, where biased predictions can lead to health disparities. Like other medical AI systems, CAC detection models may inherit biases from training data that disproportionately represent certain demographics, potentially affecting accuracy for underrepresented groups [45]. For instance, a model trained primarily on data from a specific geographic region or patient population may perform poorly in other settings, leading to inaccurate risk assessments.

A recent study by Seyyed-Kalantari et al. investigated racial and ethnic disparities in AI-driven medical imaging models, revealing that algorithms often underperform in minority groups due to insufficient diversity in the training data [46]. In the context of CAC detection, such biases could result in disparities in cardiovascular risk stratification, with underrepresented groups potentially receiving less accurate or less timely preventive care. To address this, model developers must incorporate diverse datasets that account for variations across race, gender, age, and socioeconomic status. Additionally, employing fairness metrics [47] in AI development can help quantify and mitigate biases, fostering more equitable AI applications in healthcare.

Regulatory and Legal Frameworks

The legal landscape surrounding AI in healthcare is complex, with questions of accountability and liability in the event of AI errors or misdiagnoses [48]. If an AI model incorrectly classifies a patient’s CAC level, leading to delayed or inappropriate intervention, determining liability can be challenging. For AI-driven CAC detection to be safely integrated into clinical workflows, clear guidelines on accountability are necessary, specifying whether responsibility lies with the technology provider, the healthcare institution, or the overseeing clinician.

The Food and Drug Administration (FDA) has taken steps toward regulating AI in medical devices, implementing a framework for software as a medical device (SaMD) to monitor the efficacy and safety of AI-driven tools [49,50]. In 2021, the HealthCCSng algorithm for incidental CAC detection was granted FDA clearance, marking a significant step in regulatory recognition of AI for cardiovascular care [51,52]. This clearance signifies that AI algorithms for CAC detection must meet rigorous safety standards, including transparency in algorithm development, ongoing monitoring, and post-market surveillance to ensure reliable performance across various clinical settings.

Transparency and Patient Communication

Incorporating AI into medical diagnostics also raises concerns about transparency and patient communication. Unlike traditional diagnostic methods, AI algorithms function as "black boxes," often providing little insight into how decisions are made [53,54]. Transparency in AI-based healthcare tools is crucial for fostering patient trust. When patients understand how AI models function and how their results impact health decisions, they are more likely to trust clinicians and engage in shared decision-making with the clinicians in their healthcare journey [55]. Effective communication strategies are essential, especially in incidental CAC detection, where patients may not expect cardiovascular findings on noncardiac imaging. Clinicians should be equipped to discuss the role of AI in detecting CAC, including its benefits and limitations, to ensure that patients feel informed and empowered to make decisions regarding their care.

Balancing Innovation with Ethical and Legal Safeguards

The rapid development of AI in healthcare necessitates a balanced approach that encourages innovation while maintaining stringent ethical and legal safeguards. As CAC detection algorithms become increasingly sophisticated, ongoing monitoring and updates will be essential to ensure they remain accurate, unbiased, and effective across diverse patient populations. Collaboration among AI developers, healthcare providers, regulatory bodies, and ethicists will be critical to establishing best practices for AI implementation that prioritize patient safety, equity, and privacy.

A recent review by Rajpurkar et al. on the ethical and regulatory aspects of AI in cardiology underscores the importance of integrating these safeguards at every stage of AI development and deployment. The authors advocate for multistakeholder oversight and proactive policies to address ethical challenges as AI continues to shape the future of cardiovascular care [56]. By embracing these recommendations, healthcare systems can harness the full potential of AI-driven CAC detection while upholding the ethical principles essential to patient-centered care.

Research gaps and future directions

Although AI-driven CAC detection on noncardiac CT scans has made significant strides, several research gaps must be addressed to ensure its widespread adoption, accuracy, and efficacy in preventive cardiology. Addressing these gaps will not only enhance the reliability of AI-based CAC detection but also facilitate its seamless integration into diverse clinical settings.

Standardization in CAC Quantification Across Imaging Protocols

One of the primary challenges in AI-driven CAC detection is the lack of standardized thresholds and protocols for quantifying CAC across different CT scan types [57,58]. CAC scoring protocols in cardiac imaging are well-established [59]; however, when applying AI models to noncardiac CT scans, variations in scan resolution, field of view, and slice thickness can influence CAC measurements, impacting risk assessment accuracy [57,60]. Future research should focus on establishing universally accepted CAC quantification standards for incidental detection, with guidelines that adjust for imaging variations across patient populations and clinical settings.

Broader Validation Across Diverse Populations

It is crucial for effective cardiovascular risk assessment that AI-driven CAC detection models are accurate across diverse populations. However, many existing AI models have been developed and validated using data from limited demographic groups, which can affect their generalizability. For instance, a study by Ihdayhid et al. developed an AI-based model for automated CAC scoring and tested it on a dataset with a mean age of 55.7 ± 10.5 years and 49% male participants [60]. While the model showed high accuracy, the study did not provide detailed information on the ethnic diversity of the cohort, leaving questions about its applicability across different racial groups.

To improve the generalizability of AI-driven CAC detection, it is essential to conduct validation studies that include participants from various racial, gender, age, and health condition backgrounds. This approach will help ensure that AI models provide accurate cardiovascular risk assessments for all individuals, regardless of their demographic characteristics.

Integration of AI-Based CAC Detection Into Radiology Workflows

AI models must function seamlessly, generating assessments with minimal disruption, to integrate AI-driven CAC detection effectively into radiology workflows. The NOTIFY-1 trial demonstrated success with automated notifications to primary care providers, facilitating timely patient care [29]. Future research should explore real-time detection systems, radiologist feedback mechanisms, and user-friendly methods for presenting AI findings to enhance clinical utility while reducing radiologist workload.

Reducing False Positives and Improving Specificity

While AI-driven CAC detection models have demonstrated high sensitivity, they sometimes generate false positives, especially when calcifications in other anatomical structures are misinterpreted as CAC [36]. Reducing these false positives is crucial to preventing unnecessary follow-ups, alleviating patient anxiety, and optimizing healthcare resources. Future research should focus on integrating anatomical contextual information and continuously refining AI models to improve precision in CAC detection.

## Conclusions

AI-driven incidental CAC detection on noncardiac CT scans offers a transformative pathway for broadening cardiovascular risk assessment, particularly in populations not typically undergoing cardiac imaging. By leveraging AI to analyze routine imaging studies, clinicians can opportunistically identify patients with subclinical atherosclerosis, facilitating earlier interventions that may improve outcomes and optimize healthcare resources. This approach enhances accessibility and cost-effectiveness, aligning with preventive medicine’s goals to reduce the burden of cardiovascular disease.

Despite its promise, AI-driven CAC detection presents challenges in data privacy, model bias, and regulatory oversight. Addressing these issues, alongside efforts to standardize protocols, validate across diverse populations, and link findings to clinical outcomes, will be essential to its responsible and effective integration into clinical workflows.

With continued advancements, ethical safeguards, and interdisciplinary collaboration, AI-driven CAC detection can become a cornerstone of preventive cardiology. It can make early cardiovascular risk stratification more accessible and ultimately contribute to better population health.
